# Nutrition and Stimulation

**Published:** 1858-04

**Authors:** 


### NUTRITION AND STIMULATION

Is the subject of an editorial in No. 176 of *Life Illustrated*, which, for its practical importance, cannot well be over-estimated. Many measure their health by the amount of food they can swallow. Some think that they will be well if they can only "get up an appetite;" and to accomplish that, they begin to guzzle bitters, wine, brandy, aqua-fortis mixtures, gin, rum, and poor whisky, which, for its speedily destructive effects on the intestinal canal, is truthfully, although rudely described, as *Rot-gut.*

It ought to be remembered that, as a very general rule, when there is no appetite, it is kindly Nature's warning that the system needs no nutriment—that it requires rest—and, for that very reason, takes away all desire for food; and, in three cases out of four, if persons who are conscious of not feeling well, and have no appetite, would simply avoid eating anything, rest comfortably warm in bed, and walk moderately out of doors—moderately as to gait, time, and distance, a more radical cure would result, if there is a return to eating, only as the appetite calls for it, than can be effected in any other way.

But suppose an appetite is waked up by the liquors drunk, a more decided call for food may be the result; but not an atom of additional power of digestion is given to the stomach; hence the amount of stomach-work is increased, without any increase of ability to perform that increased task. Hence persons are weeks and months taking "bitters," before they get well, and by that time they have become so much accustomed to the stimulant that it cannot be conveniently discontinued, and a dram-drinker for life is made; and, in multitudes of instances, the result is moral, social, financial, and physical death in the gutter, if not in the poor-house.

The only safe stimulant is plain, substantial food—fresh, perfect, and well-prepared. When that stimulant is needed by the body, Nature calls for it with unerring certainty—that call is *Hunger*—she may be forced to make that call, by the use of stimulating drinks; but the "transaction" never passes with impunity. Let man, then, imitate the brute creation, by eating only when he is hungry, and drinking only when he is dry.

